# Improving access to primary care for Aboriginal babies in Western Australia: study protocol for a randomized controlled trial

**DOI:** 10.1186/s13063-016-1206-7

**Published:** 2016-02-12

**Authors:** Daniel McAullay, Kimberley McAuley, Rhonda Marriott, Glenn Pearson, Peter Jacoby, Chantal Ferguson, Elizabeth Geelhoed, Juli Coffin, Charmaine Green, Selina Sibosado, Barbara Henry, Dorota Doherty, Karen Edmond

**Affiliations:** University of Western Australia, 35 Stirling Highway, Crawley, WA 6009 Australia; Edith Cowen University, 2 Bradford St, Mount Lawley, WA 6050 Australia; Murdoch University, 90 South St, Murdoch, WA 6150 Australia; Telethon Kids Institute, 100 Roberts Rd, Subiaco, WA 6008 Australia; Western Australia Department of Health, 189 Royal Street, East Perth, WA 6004 Australia; Geraldton Regional Aboriginal Medical Service, Holland St, Geraldton, WA 6530 Australia; Geraldton Regional Hospital, 51-85 Shenton St, Geraldton, WA 6530 Australia; Derbarl Yerrigan Aboriginal Medical Service, 156 Wittenoom St, East Perth, WA 6004 Australia; King Edward Memorial Hospital, 374 Bagot Rd, Subiaco, WA 6008 Australia; Princess Margaret Hospital for Children, Roberts Rd, Subiaco, WA 6008 Australia

**Keywords:** Primary care, Aboriginal, Care coordination, Infants, Intervention, Health services

## Abstract

**Background:**

Despite a decade of substantial investments in programs to improve access to primary care for Aboriginal mothers and infants, more than 50 % of Western Australian Aboriginal babies are still not receiving primary and preventative care in the early months of life. Western Australian hospitals now input birth data into the Western Australian electronic clinical management system within 48 hours of birth. However, difficulties have arisen in ensuring that the appropriate primary care providers receive birth notification and clinical information by the time babies are discharged from the hospital. No consistent process exists to ensure that choices about primary care are discussed with Aboriginal families.

**Methods/Design:**

We will undertake a population-based, stepped wedge, cluster randomized controlled trial of an enhanced model of early infant primary care. The intervention is targeted support and care coordination for Aboriginal families with new babies starting as soon as possible during the antenatal period or after birth. Dedicated health professionals and research staff will consult with families about the families’ healthcare needs, provide information about healthcare in the first 3 months of life, offer assistance with birth and Medicare forms, consult with families about their choice for primary care provider, offer to notify the chosen primary care provider about the baby’s health needs, and offer assistance with healthcare coordination at the time of discharge from the hospital.

We will evaluate this model of care using a rigorous stepped wedge approach. Our primary outcome measure is a reduced hospitalization rate in infants younger than 3 months of age. Secondary outcome measures include completed Aboriginal and Torres Strait Islander child health screening assessments, immunization coverage, and satisfaction of the families about early infant primary care. We will also assess the cost effectiveness of the model of care.

**Discussion:**

This study will be conducted over a 4-year period in partnership with birthing hospitals and primary care providers including Western Australian Aboriginal Community Controlled Health Services and the new Primary Health Networks. The results of our trial will be used to develop improved primary care models and to improve health outcomes for all Aboriginal infants. These are vital steps toward more equitable health service delivery for the Aboriginal and Torres Strait Islander children in Australia.

**Trial Registration:**

Australian New Zealand Clinical Trials Registry

Registration number: ACTRN12615000976583

Date registered: 17 September 2015

## Background

The health of many Australian Aboriginal infants continues to be poor despite substantial investments from federal and state governments [1, 2]. The early infant period (from birth to 3 months of age, 0 to < 3 m) is the period when babies are most vulnerable, have the highest hospitalization rates, and are most in need of primary care services [3, 4]. Australian Aboriginal babies are scheduled for six contacts with primary care providers during this period for immunization (diphtheria, tetanus, pertussis (DTP), hepatitis B, Haemophilus influenzae type b, poliovirus, rotavirus, and pneumococcal vaccines) and for preventive care (screening for newborn blood spot, hearing, growth, development, oral health, ear disease, skin infections, family health and wellbeing, and the medical “6-week check”) [5, 6].

However, concern is increasing in the Aboriginal community that despite high-quality hospital-based maternity services, more than 50 % of Western Australian (WA) Aboriginal babies are not receiving the recommended scheduled primary care in the early infant period [2, 7]. More than 50 % of Aboriginal babies have not received Medicare registration in the first 7 days of life, and 40 % of the infants are overdue or have not received basic hepatitis B and DTP immunizations [8, 9]. Hospital admissions are twofold higher in Aboriginal than in non-Aboriginal babies during this period, and emergency department presentations are even higher [10–12].

Primary care services (that is, the first level of community healthcare) have the potential to have a major impact on the health of young Aboriginal infants during the early infant period [5, 7]. Community child-health nurses are considered the traditional providers of infant care during this period. However, general practitioners, practice nurses, and Aboriginal health workers at Aboriginal Community Controlled Health Services (ACCHSs), government health clinics, and general practice surgeries have been used by more than 90 % of Aboriginal families during the early infant period. These health professionals are not seen as traditional providers of early infant care, and many of these staff members lack the appropriate confidence and skills for managing young infants [13, 14]. Few of these nontraditional early infant care providers routinely receive birth notification information due to concerns from government departments about confidentiality and consent. Information is often provided using ad hoc systems including facsimile machines. In addition, few primary care providers receive the education, training, and tools needed to assist them in managing young infants. Primary care providers also often receive insufficient up-to-date information on the mother’s current concerns and needs and are unable to provide assistance with coordination of appointments and transport. This is especially the case for families who are most mobile; who move between regions, districts, and jurisdictions; and families who have not been able to connect with primary care providers [15–17].

New models of early infant primary care provision (for example, home visiting models, family-centered primary health care, and educational interventions) have been extensively evaluated and are highly efficacious in improving child health and developmental outcomes in Australia and internationally [18–22]. However, these programs involve the provision of new services and have problems with scale-up, cost effectiveness, and sustainability. The programs have also not been able to overcome the difficulties of maintaining contact with the most disadvantaged families who move between jurisdictions, the sharing of personal information with other primary care providers, and the engagement and communication between primary care services, especially in areas where families use multiple primary care service providers. New personally controlled electronic health records may assist with these issues but are new in implementation, and concerns exist that they will not benefit the most marginalized and disadvantaged families [17].

Personalized targeted support and care coordination from maternity hospitals has great potential to reduce barriers in provision of early infant primary care [23–26]. However, the application of current models has been limited in Australia and internationally due to restrictions within regions and jurisdictions. Models have also not been tested in complex environments with multiple nongovernmental organizations and primary care providers. Existing systems also continue to struggle with the provision of care to the most mobile families and have not tried to improve choice in primary care providers to families or assess the effect on access and utilization of services and health outcomes during the early infant period [25, 26].

The WA Department of Health (DOH) electronic clinical management system was developed in 2005 [27]. In 2013, all WA hospitals began inputting birth data into this clinical management system within 48 hours of birth and data are now being provided to government health services within 48 hours of birth. However, difficulties exist in ensuring that the appropriate primary care providers receive birth notification and clinical information by the time the babies are discharged from the hospital, and no process is currently in place to ensure that choices in primary care are discussed with Aboriginal families.

We have thus developed a population-based, stepped wedge, cluster randomized trial to test the effectiveness of an enhanced model of targeted support and early infant primary care coordination to improve access to primary care and the health of Aboriginal infants under 3 months of age in WA.

The primary outcome measure is a reduced hospitalization rate in infants aged younger than 3 months. Secondary outcome measures include completion of child health screening assessments, immunization coverage, and satisfaction of the families about early infant primary care. We will also assess the cost effectiveness of the model of care.

## Methods

### Study setting

This trial will be conducted across all of WA, which has a population of 2.5 million people and covers a geographical area of 2.5 million km^2^ [28]. WA has the most remote regions in Australia, with 95 % classified as ARIA (Accessibility/Remoteness Index of Australia) level 4 (remote) and 5 (very remote). Approximately 40 % of the Indigenous WA population resides within these remote and very remote regions of WA [28]. In 2010, there were 29,160 births in non-Aboriginal and 2,013 births in Aboriginal women [27]. Identification of Aboriginal and Torres Strait Islander ethnicity is improving in records of hospitalizations, with 96 % being correctly identified in 2011 to 2012 [29]. Thirty-seven birthing hospitals are located in WA: 28 public (one tertiary maternity hospital, and 27 secondary hospitals) and nine private birthing hospitals.

Approximately 2,900 primary healthcare services currently exist in WA, including 20 Aboriginal Community Controlled Health Services (ACCHS), which provide health services to regional, rural, and remote communities across WA, as well as the metropolitan areas. Nearly 400 government community health clinics and more than 2,000 registered general practice clinics exist. All births are attended by a registered midwife who must complete a birth registration form within 48 hours of birth. The state has been divided into 22 distinct areas separated by geographic, tribal, and health service provider boundaries, which provide the basis for our clusters.

This study is being coordinated from the Princess Margaret Hospital for Children (PMH) in Perth.

### Trial design

**Fig. 1:**
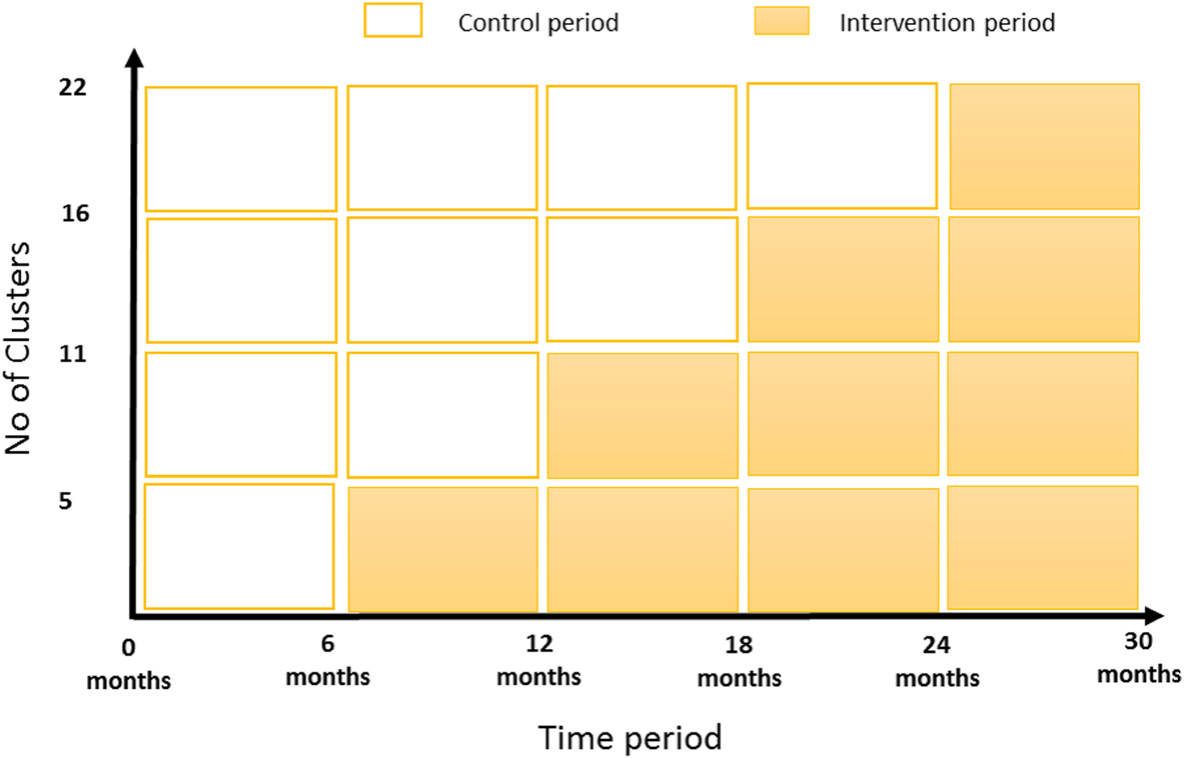
Stepped wedge, cluster randomized controlled trial study design. Each cell represents five or six clusters, and a data collection point. Twenty-two clusters with approximately 4,275 birth notifications will be assessed for eligibility, randomized, and allocated to the intervention or control groups. At the time point 0 months, the baseline evaluation will occur. At the time point 6 months, five clusters will be randomized to receive the intervention. By the time point 24 months, all clusters will be receiving the intervention

**Fig. 2:**
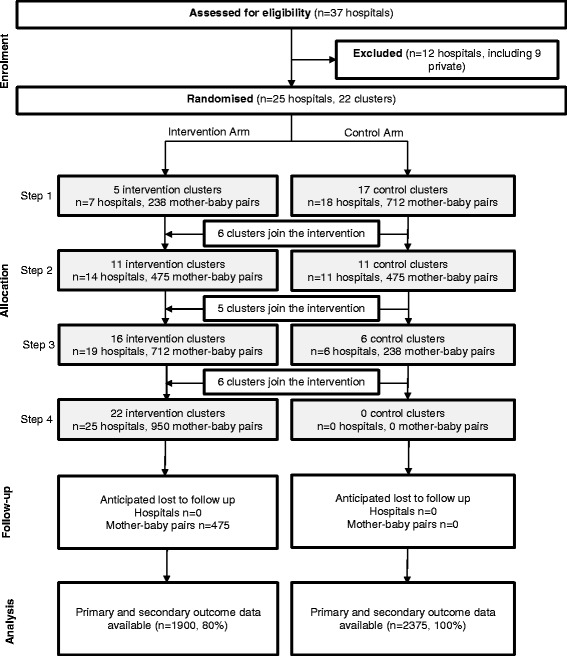
Trial profile

This will be a stepped wedge, cluster randomized controlled trial (RCT) (Fig. 1). This is one of the most appropriate study designs for evaluating health system interventions [30]. Advantages include enhanced motivation from patients and health professional staff to participate because all clusters receive the intervention, the design is more ethical with quality improvement initiatives that are likely to involve more good than harm within the study population, and the study can reduce the required number of clusters and therefore requires less financial input [30, 31]. The intervention effect is estimated by the between-cluster (those awaiting the intervention and those receiving the intervention) and within-cluster (before and after) comparisons. All public birthing hospitals in WA have been grouped into 22 distinct geographic clusters and will be randomized in a “stepped” (staggered) order to receive our model of targeted early infant primary care or to continue with standard care. The trial will consist of a baseline period of 6 months, followed by four steps of 6-month duration. Five or six new clusters will receive the intervention at each step, and all hospitals and primary care clinics will receive the intervention by the end of the second year of the trial. The CONSORT cluster RCT and pragmatic trial guidelines will be followed [32, 33]. The trial profile is outlined in Fig. 2. The trial has been registered in the Australian New Zealand Clinical Trials Registry (ANZCTR) (Registration number ACTRN12615000976583).

### Inclusion and exclusion criteria

Thirty-seven birthing hospitals are located in WA; 28 public (one tertiary maternity hospital and 27 secondary hospitals) and nine private birthing hospitals. All public birthing hospitals in WA with five or more annual births of Aboriginal babies will be invited to participate in the intervention. All Aboriginal mothers who give birth to a live baby will be invited to participate in the study. Mothers will be asked for their written informed consent to be part of the study and to have evaluation data collected. We will not exclude any women who deliver a preterm baby, babies who develop complications, or any unwell babies, as these babies are most likely to benefit most from the improved skills of their primary care workers.

### Blinding

All intervention and control clusters will be blinded to the specific process outcomes that will be evaluated. However, blinding the clusters to the intervention and to the clinical outcomes will not be possible because they will be aware of the study protocol.

### Development of project tools

We will first develop generic guidelines, protocols, and tools for all components of the intervention: hospital consultation, families, and primary care providers. We will test and adapt all tools during a pilot phase in one pre-trial maternity hospital.

### Randomization

A stepped wedge, cluster randomized controlled trial design was chosen in which hospitals are allocated into geographical clusters and then randomized into two groups (intervention or control) within each of the five steps (Fig. 1). Twenty-five birthing hospitals included in the sampling frame have been grouped into 22 geographical clusters defined by postcode. As such, these clusters are the unit of randomization in this trial. The cluster randomization was completed using a random number generator in Excel. A member of the research team grouped the 25 hospitals into 22 clusters and labelled these clusters 1 to 22. A separate Excel spreadsheet was created with a column containing the numbers 1 to 22. No information about the corresponding hospitals was present in this document. The de-identified Excel file was given to our statistician, who was blinded to the corresponding cluster information. The random number generator was used to generate a number from 0 to 1 next to each number in the column containing the de-identified cluster data (numbers 1 to 22). The two columns were then sorted by the column containing the random number, from the smallest to the largest. Five steps are included in this stepped wedge design, each representing 6 months. During the first step, or the first 6 months, all clusters are controls. Allocation into the intervention or control group for the additional four steps is as described below. Using the sorted list provided by the statistician, the first five clusters were allocated to the intervention group in step 1. Once allocated to the intervention group, a cluster continues in the intervention group for the remainder of the trial. The remaining 17 clusters are allocated to the control group for step 1. For step 2, the six clusters that immediately follow the first five clusters in the list were allocated to the intervention group. In total, 11 clusters were allocated to the intervention group by step 2. The remaining 11 clusters were allocated to the control group. The next five clusters in the random number generator sorted list were allocated to the intervention in step 3. The remaining six clusters were allocated to the control group. The final six clusters were allocated to the intervention group in step 4. The intervention commencement will be staggered at 6 monthly intervals, and all clusters will receive the intervention by step 4.

### Sample size calculation

The proposed sample size calculation is based on our primary hypothesis that the new model of care will result in a significant reduction in all-cause hospitalization in infants aged 0 to < 3 months of age. Our pilot data indicate that the approximate number of Aboriginal babies delivered in the birthing hospitals included in the sampling frame is 1,900 per year. We therefore anticipate approximately 4,750 Aboriginal births during the 30-month trial period. Of these, approximately 2,375 will be born in the control setting, and 2,375 in the intervention setting. All families delivering within the hospitals implementing the intervention will have the opportunity to participate in the trial. We estimate a 20 % refusal and loss to follow-up rate within the intervention group. Thus, we anticipate approximately 1,900 Aboriginal infants born over a 30-month period will be involved in the intervention. In total, approximately 4,300 Aboriginal infants will be followed up in the trial. Twenty-five hospitals with maternity units in WA comprise 22 distinct geographical clusters, with an average of 39 anticipated births per cluster per 6-month step. The average current prevalence of any (all cause) hospitalization in infants aged 0 to < 3 months is 31 %. Early data indicate that hospitalization rates range from 15 to 50 % across the clusters. Estimation of the study power requires calculation of the design effect, which, in the context of a stepped wedge design, is the factor which must be applied to the total number of subjects required in a similarly powered individual-level parallel group design to give the number of subjects per step in a stepped wedge design. We calculated the intracluster correlation (ICC) based on previous hospitalization rates. The calculated ICC of 0.017 was used in the Woertman et al. [28] formula to calculate the design effect with the outcome being 0.57. With 4,300 babies followed up over the 30-month period, the study will provide 80 % power to detect, at a significance level of *P* < 0.05, a reduction in prevalence of any early hospitalization of 31 % to 24.5 % because of the intervention.

### The intervention group

The targeted support and care coordination intervention is outlined in Table 1. Initially we will work with birthing hospitals to understand gaps in infant care coordination and target project tools to meet local needs. These consultations will inform the specifics of the care coordination activities, which will include the following:

**Table 1:** Description of the intervention

The following three components of the intervention will be implemented by dedicated infant healthcare coordinators:
Hospitals - Work with birthing hospitals to understand gaps in infant care coordination and target project tools to meet local needs.
Families - Meet families as soon as acceptable to the families after birth. Use the locally targeted care coordination tools for the following:
- consult with families about their health care needs,
- provide information about primary care services available for the first 3 months of the baby’s life,
- offer assistance with birth and Medicare registration forms,
- consult with families about their choice for primary care provider,
- offer to notify the chosen primary care provider about the baby’s health needs, and
- offer assistance with health care coordination at the time of discharge from hospital.
Primary care providers - Work with the family’s chosen primary care provider to achieve the following:
- ensure they receive birth and hospital details,
- offer tools and guidance on early infant primary care, and
- offer additional assistance and contact points with experienced community child health nurses, primary care networks and pediatricians.

1. Assessing the needs and goals by consulting with families about their healthcare needs, their preferred primary care provider, and assistance with Australian Government forms (Medicare and birth registration)
2. Facilitating transitions across settings by providing information to the families about the primary care services available for the first 3 months of the baby’s life, offering to contact the chosen primary care provider on behalf of the family, offering assistance with healthcare coordination at the time of discharge; and by offering additional assistance to primary care providers and contact points with experienced community child health nurses, primary care networks, and pediatricians
3. Communication through interpersonal interactions with the families and primary care providers, and transferring information through direct contact between the hospital and chosen primary care provider, ensuring they receive birth and hospital details

Coordination activities will be led by dedicated infant healthcare professionals, who will meet with families as soon as acceptable after birth. Mothers will be asked for their written informed consent to be part of the study and to have evaluation data collected.

### The control group

The control clusters will be the hospitals and clinics located in clusters that have not yet received the intervention. The control clusters will receive standard care according to regional guidelines without additional support. The control clusters will receive the same level of evaluation data collection as the intervention groups.

### Data collection

Outcomes will measure the impact of the intervention from a systems perspective, including the quality of care, healthcare utilization and cost. Outcomes will also measure the process of the intervention from family and healthcare professional perspectives.

Information from routine data capture systems (midwives notification system, hospital morbidity database, emergency department database, Australian Immunisation Register (ACIR) database, and the Commonwealth Medical Benefits System (MBS) database) will be collected from both the control and intervention groups. These data will include the following: (i) sociodemographic data: obstetric history, age, sociodemographics, medical history, morbidities, birth weight, gestational age, Apgar score, and discharge date; (ii) hospitalization data from all WA hospitals on all-cause hospitalization and emergency department presentations; (iii) immunization data from ACIR (date of first and second doses of hepatitis B and date of first dose of DTP containing vaccine) from birth until the baby reaches 3 months of age; and (iv) Medicare data from the MBS (Date of Medicare registration and dates of child health checks (item number 715)) from birth until the baby reaches 3 months of age.

Economic evaluation will compare outcomes between the intervention and control groups. Consequences will be measured as the health gains from the child health assessments and immunizations received. Short-term health gains include reduced emergency department presentations and hospital admissions during the first 3 months of life. Long-term health gains include improved child neurodevelopment (especially speech delay, concentration, learning and educational outcomes) and reduced infectious disease (including gastroenteritis, Haemophilus influenzae type b (Hib), pneumococcal disease and pertussis).

Some data will be collected only in the intervention clusters. This will include questions from structured questionnaires querying the mother about her satisfaction with the healthcare she received during the antenatal and postnatal periods. Process data will also be collected on the needs and support required by the hospitals and primary care providers including IT resources, formal education sessions, guidelines and protocols distributed, and telephone calls. Economic data will include the full cost of implementing the intervention in each cluster. This will include the costs of the new intervention including all aspects of delivery, tools, training visits (including vehicle hire, petrol, accommodation, meals) telephone calls, and dedicated staff salaries.

### Data analysis

Data analysis will be conducted on the basis of intention to treat, regardless of movement between clusters. The intervention effect will be estimated by the between-cluster (those awaiting the intervention and those receiving the intervention) and within-cluster (before and after) comparisons. An analysis of the primary outcome (any hospitalization in the first 3 months of life) will be conducted at the individual infant level and will use binary logistic regression incorporating generalized estimating equations (GEE) to account for the loss of independence due to clustering. The primary independent variable will be whether or not the baby received the intervention. This will give rise to an odds ratio for the effect of the intervention on the probability of early hospitalization. The analysis will adjust for individual covariates, for example, maternal age, which may differ between groups receiving or not receiving the intervention at a given step. Similar techniques will be used for comparison of emergency department presentations and immunization coverage.

A societal viewpoint will be taken for the economic evaluation where all costs and consequences to the mothers and the wider community will be considered. All direct (fixed and variable) costs will be considered and will include the monetary costs of the intervention and resources. Consequences will be measured as hospitalizations averted. Incremental direct costs will be calculated by comparing the costs generated by the intervention with the costs generated by the control arm of standard care. Incremental consequences will be calculated by comparing the number of hospitalizations in the intervention clinics with the number of hospitalizations in the control clinics. Incremental cost-effectiveness ratios (cost per infant hospitalization case averted) will be calculated by dividing the incremental direct costs by the incremental hospitalization cases averted. Sensitivity analysis (a method to determine the robustness of an assessment by examining the extent to which results are affected by changes in methods, values of variables or assumptions) will also be performed. Satisfaction with care will also be assessed descriptively. The process outcomes and implementation of the intervention will also be assessed descriptively.

### Ethical issues

This research will be conducted in accordance with the NHMRC National Statement on Ethical Conduct in Human Research and Guidelines for Ethical Conduct in Aboriginal and Torres Strait Islander Health Research. The protocol for this study has been approved by the Western Australian Aboriginal Health and Ethics Committee (WAAHEC), The University of Western Australia Human Research Ethics Committee and the Western Australian Country Health Service Human Research Ethics Committee. All mothers in the intervention clusters will be asked for their written informed consent to be part of the study and to have evaluation data collected. In the control clusters, we will not have the opportunity to take individual consent from mothers. However, we will apply to the data linkage branch of WA Health for the routine data capture of system data without identification.

## Discussion

Many studies have described the problems with primary care services, such as screening and immunization, for Australian Aboriginal and Torres Strait Islander children without attempting to test potential solutions. This will be the first population-based trial to investigate the use of early infant primary care coordination to improve access to primary care and infant outcomes.

Our study is both region- and population-based and is a cluster randomized controlled trial. This will limit the problems of selection bias and confounding present in other published studies. Our sample size of 4,300 births will provide sufficient power to detect clinically important effects for all primary and secondary outcomes. The potential exists for contamination from other clusters due to the migration of women. However, our clusters are geographically defined and grouped by care-seeking patterns and skin and language group. Contamination of the health service provider education and training across clusters is also possible. However, health provider training will also be kept distinct until the final health service has been recruited into the trial.

Our intervention is embedded within the health system and is likely to be both cost effective and sustainable. Our study is directed by our experienced Aboriginal chief investigators. Thus, our study will have important cross-cultural relevance and impact. The results of our study will be used to develop improved primary care models and to improve health outcomes for Indigenous mothers and infants and other vulnerable populations. These are vital steps toward more equitable health service delivery for Aboriginal and Torres Strait Islander and other disadvantaged populations.

## Trial status

Recruitment will commence in 2016.

## Abbreviations

ACCHS: Aboriginal Community Controlled Health Service
ANZCTR: Australian New Zealand Clinical Trials Registry
CONSORT: consolidated standards of reporting trials
DOH: Department of Health
DTP: diphtheria, tetanus, pertussis
GEE: generalized estimating equations
PMH: Princess Margaret Hospital for Children
RCT: randomized controlled trial
WA: Western Australian
WAAHEC: Western Australia Aboriginal Health and Ethics Committee
